# Microbial Carbonic Anhydrases in Biomimetic Carbon Sequestration for Mitigating Global Warming: Prospects and Perspectives

**DOI:** 10.3389/fmicb.2017.01615

**Published:** 2017-08-25

**Authors:** Himadri Bose, Tulasi Satyanarayana

**Affiliations:** Department of Microbiology, University of Delhi New Delhi, India

**Keywords:** global warming, polyextremophilic microbes, thermo-alkali-stable, carbonic anhydrase, biomineralization, CA inhibitors

## Abstract

All the leading cities in the world are slowly becoming inhospitable for human life with global warming playing havoc with the living conditions. Biomineralization of carbon dioxide using carbonic anhydrase (CA) is one of the most economical methods for mitigating global warming. The burning of fossil fuels results in the emission of large quantities of flue gas. The temperature of flue gas is quite high. Alkaline conditions are necessary for CaCO_3_ precipitation in the mineralization process. In order to use CAs for biomimetic carbon sequestration, thermo-alkali-stable CAs are, therefore, essential. CAs must be stable in the presence of various flue gas contaminants too. The extreme environments on earth harbor a variety of polyextremophilic microbes that are rich sources of thermo-alkali-stable CAs. CAs are the fastest among the known enzymes, which are of six basic types with no apparent sequence homology, thus represent an elegant example of convergent evolution. The current review focuses on the utility of thermo-alkali-stable CAs in biomineralization based strategies. A variety of roles that CAs play in various living organisms, the use of CA inhibitors as drug targets and strategies for overproduction of CAs to meet the demand are also briefly discussed.

## Introduction

In the early Eighteenth century, industrial revolution took the world by storm. This led to large scale manufacture, which at that time proved to be a major economic boost world over. In the last 300 years, there has been a marked transformation in human life. Due to improved farming practices, food production increased. This along with technological advances in health, communication and transport sectors paved the way toward modern age for human civilization. This modernization led to a heavy toll on “mother nature” (Shakun et al., 2012).

The concentrations of greenhouse gases are increasing day by day mainly due to anthropogenic activities, of them about two-thirds is contributed by fossil fuels. The burning of fossil fuels results in the emission of large quantities of flue gas that contains ~ 71% N_2_, 14% CO_2_, 1–2% hydrocarbons, carbon monoxide, NO_x_, and minor amounts of SO_x_ (Perry and Gee, 1995). According to IPCC, among all the greenhouse gases, CO_2_ is emitted most (65% from fossil fuels and 11% from forestry). Other gases such as methane (16%), nitrous oxide (6%), and fluorinated gases (2%) are emitted in smaller amounts by anthropogenic activities (IPCC, 2014). Cumulative carbon emissions from different sectors have increased by about 40% since 1970s. The concentrations of hazardous gases such as NOx, SOx and methane are well beyond their threshold in many cities all over the world, thus, the Air Quality Index (AQI) is declining (IPCC, 2000). CO_2_ levels in the atmosphere have surged past the threshold of 400 ppm and it may not climb down for generations. This 400 ppm benchmark was broken first time in the recorded history last year. According to World Meteorological Organization (WMO), 2016 would be the first full year to exceed the mark (NOAA, 2016). As per the latest measurement by NOAA in April 2017, the concentration of CO_2_ at present is 406.17 ppm (NOAA, 2017). Some of the isolated places (Arctic regions) have already breached this mark in the past few years as recorded in Mouna Loa Observatory in 2013 (IPCC, 2013). Emission Database for Global Atmospheric Research stated that global emission of CO_2_ has increased by 48% in the last two decades (http://edgar.jrc.ec.europa.eu/overview.php?v)^1^. The increase in GHG emissions has led to increase in earth's surface temperature by about 2°C from pre-industrial times (IPCC, 2000). These conditions have also led to widespread natural calamities and affected the environment adversely. An increase in warm temperature extremes and decrease in cold temperature extremes have been noted in the past few years. This has also led to an impact on the precipitation patterns around the world and disturbed the water cycle. Agricultural production has been affected due to adverse climatic changes. There has been a reduction in crop yields leading to increase in food prices, food shortage and insecurity (Adams et al., 1998). These climatic hazards are affecting the lives of people round the world particularly those who are living below the poverty line. Many freshwater, marine and terrestrial species are already on the verge of extinction. A change in the distribution and interaction pattern has been observed in many freshwater and marine phytoplanktons. According to IPCC, the period from 1983 to 2012 were the warmest years (IPCC, 2014).

Air pollution causes various respiratory and cardiac diseases. Tiny particles produced by vehicular engines and industry worsen heart and brain related disorders and increase the risk of stroke. Global warming is taking the earth toward peril and it is essential to tackle this catastrophe for our survival. It is next to impossible for developing and under developed nations to control large scale CO_2_ emissions. Nevertheless, global warming has to be mitigated. Scientific and global consensus on global warming and climate change has brought the world powers together in order to hunt for new technologies for mitigating the global warming (Kheshgi et al., 2012).

The year 2015 ushered in an era of optimism and action with Paris climate change agreement. It also marks a new era of climate change reality with record levels of high greenhouse gases. In order to tackle increasing carbon emissions, carbon trading and taxation have been implemented by various countries (Princiotta, 2007). The progress made in developing carbon capture technologies has been reviewed from time to time (Boone et al., 2013; Frost and McKenna, 2013). IPCC has also published a wholesome review on different CCS technologies providing a precious input for policy makers and researchers in developing schemes for reducing GHG emissions (IPCC, 2000, 2013, 2014). Some reviews have also outlined various holistic approaches for carbon capture and also described methods for mitigating global warming. This review discusses biomimetic approach for mitigation of global warming with a special focus on utilizing nature's own catalyst, carbonic anhydrase (CA), as the biomimetic carbon sequestering agent.

## Approaches for mitigating global warming

Countries round the world have initiated a number of measures to counter the alarming rise of CO_2_ in the environment which includes use of low carbon fuels (nuclear power, natural gas etc.), increasing the use of renewal energy and applying geo-engineering approaches [afforestation and reforestation, and CO_2_ capture, storage and utilization (CCSU)]. All these approaches have their own set of merits and demerits. It is impossible to curtail the carbon emissions by using only one of the measures. The major drawback of these measures is very high capital investments as non-conventional energy sources are associated with high cost and at times they are not user friendly as well (Faridi and Satyanarayana, 2015). Among them, CCSU can reduce the carbon emissions by 80–85% by capturing CO_2_ directly from power plants and energy intensive processes such as cement industries, which are described in detail (Boot-Handford et al., 2014; Leung et al., 2014) in the following sections.

## Carbon capture and storage (CCS)

### CO_2_ capture

CO_2_ emissions are captured and separated from flue gas, transported via huge pipelines and stored permanently deep underground or reutilized for various applications. Various approaches have been designed for efficient CO_2_ capture. These approaches are basically divided into three phases:

- Pre-combustion CO_2_ capture
- Post-combustion CO_2_ capture
- Oxyfuel combustion

In pre-combustion CO_2_ capture, the fuel is treated initially before combustion as in the case of coal or natural gas. Coal undergoes gasification process resulting in the formation of syngas (CO + H_2_). The reaction takes place in low O_2_ environment. Further, this syngas (which is free from pollutants) undergoes water gas shift reaction to form CO_2_ and H_2_O. This technique can be applied in power plants using coal as a fuel source, but it incurs an efficiency loss of 7–8%. Natural gas containing mainly methane (CH_4_) can be converted to syngas and rest of the procedure is same like that of coal (Olajire, 2010). In post-combustion CO_2_ capture, carbon is captured from flue gas generated after combustion. This technique has been considered useful for the existing power plants and approved in small scale with a CO_2_ recovery rate of 800 t/day (Wall, 2007). Oxyfuel combustion uses oxygen in place of air for combustion. This substantially reduces the NOx load in the exhaust gas. Hence the remaining gas only contains SO_2_, water and CO_2_. SO_2_ can be easily removed by precipitation or desulphurization leading to about 80–98% recovery of CO_2_. This process requires large quantities of pure O_2_ that increases overall cost of the process. The presence of a large amount of SO_2_ in flue gas would cause corrosion problems (Buhre et al., 2005; Pfaff and Kather, 2009).

CO_2_ separation from flue gas is carried out by various ways such as absorption [liquid sorbents such as monoethanolamine (MEA), diethanolamine (DEA) used to separate CO_2_], adsorption (solid adsorbent such as zeolite, activated carbon are used to bind CO_2_ on its surface), chemical looping combustion (metal oxide is used as oxygen carrier for combustion), membrane separation (allows only CO_2_ to pass through while trapping other components), hydrate based separation (flue gas exposed to high pressure of water, leading to formation of hydrates and CO_2_ gets trapped within them) and cryogenic distillation (distillation at low temperature and high pressure), thereby separating the solidified CO_2_ (Aaron and Tsouris, 2005; Knudsen et al., 2009; Bhown and Freeman, 2011).

### CO_2_ transport

Large pipelines are used in transport of CO_2_ to storage sites or to facilities where it can be reutilized. Pipelines are the most efficient and viable method of CO_2_ transport to long distances. Various factors have to be considered for the proper transport of CO_2_. Mass/volume ratio is optimized by transporting the CO_2_ in supercritical conditions (i.e., pressure is maintained at 72.1 atm and temperature around 32°C; Svensson et al., 2004). Impurities in the CO_2_ stream leads to alteration in pressure and temperature, thus, impurities must be taken care of. The formation of carbonic acid due to the presence of water leads to corrosion of the pipelines, which are mostly made of carbon steel. Large integrated network of pipelines are needed for a commercial CCS project (Aspelund et al., 2006). These large networks will help in decreasing overall length of the pipeline by 25%, thereby reducing the associated cost. The cost of transport also depends on the regional and economic conditions of a country. A cost analysis in China showed that for a mass flow of 4,000 t CO_2_/day, the use of ship tankers will cost 7.48 USD/tonne CO_2_ as compared to 12.64 USD/tonne CO_2_ for railway tankers and 7.05 USD/tonne CO_2_ for 300 km pipelines. Periodical monitoring and assessment of the pipelines is needed for efficient and regular transport (Gao et al., 2014).

### CO_2_ storage: geological storage

Carbon dioxide can be stored into rock pores 800 m below the surface in abandoned deep saline aquifers and oil or gas reservoirs. This is called geological carbon sequestration (Orr, 2009). The choice of a good sequestering site is always useful for long term carbon storage. Porous and permeable surfaces having non-potable ground water or the sites having sedimentary rock formations made of such chemicals (e.g., calcium) can react with CO_2_ leading to more stable formations, which are preferable sites for storage (Benson et al., 2005). The first large scale CO_2_ sequestration project which started in 1996 was called Sleipner, located in the North Sea where Norway's Statoil Hydro strips carbon dioxide from natural gas with amine solvents and disposed of carbon dioxide in a deep saline aquifer. CO_2_ can be transported in high pressures into nearly depleted oil/gas wells in order to extract the residual oil and gases, while the injected CO_2_ remains stored (enhanced oil recovery). CO_2_ can be injected into deep coal beds to release methane which is trapped in porous structure of coal seams (CO_2_–enhanced coal bed mehane). Deep aquifers at a depth of 700–1,000 m below ground level contain high salinity brines, which can be used to store injected CO_2_ captured from CCS process (Hart and Gnanendran, 2009).

### Deep ocean storage

Oceans are the natural sinks for CO_2_ and it can be injected into the ocean by direct injection and ocean fertilization. Oceans can take up about 2 billion metric tons of CO_2_ per annum, the amount of carbon that would double the load in the atmosphere and would increase the concentration in the deep ocean by only 2%. At depths of more than 3 km, CO_2_ gets liquefied and can easily sink to the sea floor due to its higher density than the adjoining seawater and can be stored there for a long time. Beside these, three other mechanisms have also been proposed for CO_2_ storage. Physical trapping of CO_2_ using immobilization in a gaseous or supercritical phase in geological formations causes its immobilization that leads to either static trapping in structural traps or residual gas trapping in porous structure. Chemical trapping in formation fluids (water/hydrocarbon) either by ionic trapping or by dissolution, where CO_2_ can react chemically with minerals after dissolution to form mineral trappings or it can get adsorbed on mineral surface (adsorption trapping). Hydrodynamic trapping through the upward migration of CO_2_ at very slow speed results in its trapping into the intermediate layers. In this manner, large quantities of CO_2_ can be stored (Benson et al., 2005). Polymer microcapsules composed of liquid carbonate cores, which have high surface area for controlled uptake of CO_2_, have been utilized for large scale carbon capture process. Higher adsorption of carbon dioxide was achieved and these microcapsules were found to be stable under typical industrial operating conditions (Vericella et al., 2015).

### Demerits of CCS technology

Although, CCS approaches are quite efficient, they still have certain implications. First of all the cost of the overall process is quite high. It has been estimated that CCS will lead to a rise in power tariff by about 10% in US alone. It also requires a large amount of energy as it consumes 25 percent of the power plant's output capacity. The use of MEA in capture methods may lead to corrosion, evaporative losses, generation of toxic degradation products, and may require significant energy to remove CO_2_ during sorbent regeneration (Nielsen et al., 2012; Reynolds et al., 2012; Da Silva and Booth, 2013). There are some other disadvantages associated with the post-combustion amine capture method. The equipment will be very large as compared to the small size of a coal-fired power plant. Large volumes of solvents are needed. Regeneration of solvent by heating produces toxic byproducts, which should be scrubbed and eliminated. This process also utilizes large volumes of water (Herzog, 1998). In this approach, the energy utilization increases by about 15–25%. During transport and storage of CO_2_, there is a risk of leakage. Direct injection of CO_2_ will lead to the acidification of deep ocean (Dutreuil et al., 2009) which may cause disastrous effects on marine ecosystem. That's why only geological storage of CO_2_ into deep saline aquifers and unused mines is somewhat acceptable (Shahbazi and Nasab, 2016). The CCS technology is still in pilot scale and a large scale commercialization is yet to be undertaken. Way back in 2010, Tsouris et al. (2010) pointed high costs associated with CCS technology and urged the world to direct its energies on alternative energy technology. It is also true that in near future it will be very difficult to move away from fossil fuels as energy source. Biomineralization of CO_2_ using metal oxides (MgO and CaO) with the help of ubiquitous biocatalyst carbonic anhydrase (CA) provides a cost effective and environmentally benign solution for mitigation of global warming, which will be discussed in detail in the ensuing sections.

### Mineralization of atmospheric carbon

This process mimics the mineralization process occurring in nature which is responsible for the presence of huge amounts of limestone on the surface of Earth. This is called silicate weathering. It traps the atmospheric carbon by reacting with large limestone rocks such as wollastonite (CaSiO_3_), serpentine (Mg_3_Si_2_O_5_(OH)_4_) and olivine (Mg_2_SiO_4_) (Huijgen et al., 2007; Santos et al., 2007). This process occurs in both salt and fresh waters as CO_2_ gets dissolved in water easily and there exists equilibrium between CO_2_, ${\text{HCO}}_{3}^{-}$, and ${\text{CO}}_{3}^{2-}$. The set of reactions involved in CO_2_ mineralization is outlined below (Farrell, 2011):

Gaseous CO_2_ dissolves quickly in water and produces a loosely hydrated aqueous form (1).

(1) $${\text{CO}}_{2}(\text{g})\to {\text{CO}}_{2}(\text{aq})$$

Then carbonic acid is formed when aqueous CO_2_ reacts with water (2).

(2) $${\text{CO}}_{2}(\text{aq})+{\text{OH}}^{-}\to {\text{H}}_{2}{\text{CO}}_{3}$$

In the 2nd step, carbonic acid breaks down into carbonate and bicarbonate ions [(3), (4)]

(3) $${\text{H}}_{2}{\text{CO}}_{3}\;\to {\text{ wHCO}}_{3}^{-}+{\text{H}}^{+}$$

(4) $${\text{HCO}}_{3}^{-}+{\text{OH}}^{-}\;\to {\text{ CO}}_{3}^{2-}+{\text{H}}_{2}\text{O}$$

The presence of metal ions such as Ca^2+^, Mg^2+^, and F^*e*2+^ drives the precipitation of carbonate into mineral carbonates as depicted below (5):

$${\text{CO}}_{3}^{2-}+{\text{Ca}}^{2+}\to {\text{CaCO}}_{3}↓(\text{calcite})$$

$${\text{CO}}_{3}^{2-}+{\text{Mg}}^{2+}\to {\text{MgCO}}_{3}↓(\text{magnesite})$$

$${\text{CO}}_{3}^{2-}+{\text{Ca}}^{2+}+{\text{Mg}}^{2+}\to \text{CaMg}({\text{CO}}_{3}{)}_{2}↓(\text{dolomite})$$

$${\text{CO}}_{3}^{2-}+{\text{Fe}}^{2+}\to {\text{FeCO}}_{3}↓(\text{siderite})$$

This process is pH dependent. At pH below 8.0, reaction 2 becomes insignificant as OH^−^ ions are absent. Between pH 8.0 and 10.0, both the reactions (2 and 3) occur, and above pH 10, reaction 2 occurs mainly. Due to abundant supply of OH^−^ at alkaline pH, mainly ${\text{HCO}}_{3}^{-}$ (bicarbonate) and ${\text{CO}}_{3}^{-}$ form leading to CaCO_3_ precipitation. Also at acidic pH, the solubility of carbonate increases. In order to increase carbonate precipitation, it is necessary to make the environment alkaline. Mineral carbonation is being studied at length for its utility in biomineralization of CO_2_ from flue gas. Some pilot scale studies have already been undertaken to demonstrate the viability of the process (Reddy et al., 2010).

This technique has several advantages over other sequestration based approaches:

1. This process is an environmentally benign and one of the most effective techniques of carbon sequestration, and carbonates produced naturally via mineralization of CO_2_ can remain stable for centuries. This process is free from complexities and many researchers have already outlined this process in minute details, hence, easily adaptable (Seifritz, 1990; Druckenmiller and Maroto-Valer, 2005; Liu et al., 2005; Stolaroff et al., 2005; Mirjafari et al., 2007; Favre et al., 2009).
2. Raw materials for mineralization of CO_2_ are in abundance. These minerals comprise a huge CO_2_ reservoir having carbon equivalent to about 150,000 × 10 metric tons of CO_2_. Metal oxides such as MgO and CaO are emitted from the industries as hazardous wastes in the form of fly ash (Soong et al., 2006). Mineral carbonation using such wastes will allow their re-utilization in sequestering CO_2_ (Stolaroff et al., 2005). Fly ash was used for mineral carbonation in USA and concentration of CO_2_ reduced from 13.0 to 9.6% and SO_2_ concentration drastically decreased from 107.8 ppm to 15.1 ppm within 2 min (Reddy et al., 2010).
3. Mineral carbonates formed after sequestration will also provide industrially valuable and useful byproducts such as cements, chemicals, fillers for paper making, white paints, and other construction materials. These mineral carbonates are also used in manufacturing calcium supplements, antacids and tableting the excipient for medical usage as well as remediation of waste feed stocks (Ciullo, 1996). Pure silica with a desirable particle size can be used as a material in the construction, plastics, electronics, and glass industries.
4. The process is economically viable, since it eliminates the large scale and energy-intensive process of solvent capture of CO_2_ from industrial wastes. This process does not require the transportation of supercritical CO_2_ into deep underground.

Despite being very effective, it has certain limitations. The process is very slow in ambient conditions (Haywood et al., 2001). According to the study of kinetics of calcite precipitation by Dreybodt et al. (1997), except at high pH, the formation of ${\text{HCO}}_{3}^{-}$ (bicarbonate) is the rate limiting step. Equilibrium constants for reactions (2) and (3) are 2.6 × 10^−3^ and 1.7 × 10^−4^, respectively (Mirjafari et al., 2007). The rate of reaction (3) and (4) is being virtually diffusion controlled and very rapid. If CO_2_ hydration rate could be enhanced in some way or the other, then maximal amount of anthropogenic CO_2_ can be converted into mineral carbonates. As it is said that the “Nature has solution to every problem,” we are endowed with a natural solution to the climate change problem in the form of carbonic anhydrase (CA). The CAs can speed up the entire mineralization process by catalyzing the hydration of dissolved CO_2_ into bicarbonate i.e., the reaction 2 at a faster rate (biomineralization). The addition of dolomite and K-feldspar to the soil can further enhance carbon sequestration in soil (Xiao et al., 2016). Use of CA as a potential biocatalyst has caught the attention of many researchers and much work has been done on exploring the possibilities of using this “Nature's own catalyst” for CCS (Farrell, 2011; Alvizo et al., 2014). Zinc(II) cyclen, which is a mimic of the enzyme carbonic anhydrase, was evaluated for its utilization in carbon capture process in rigorous conditions as that in industries and it was shown to be inhibited by bicarbonate accumulation (Floyd et al., 2013). There are some CA variants which can minimize bicarbonate inhibition by protecting the active site with a hydrophobic pocket. Hence, it is worthwhile to search for natural CA enzymes which can circumvent bicarbonate inhibition. Power et al. (2016) have successfully demonstrated the utility and efficiency of bovine carbonic anhydrase (BCA) and CO_2_-rich gas streams in the carbonation rate of brucite [Mg(OH)_2_], which is a highly reactive mineral. Carbonation was affected by decrement in CO_2_ supply. In the following sections, the role of CA in CCS has been described in greater detail.

### Carbonic anhydrase: vital cog in the wheel of life

Carbonic anhydrases catalyze CO_2_ hydration and ${\text{HCO}}_{3}^{-}$ dehydration, in almost all organisms. It (EC No. 4.2.1.1) is a zinc metalloenzyme which is used as a catalyst in living systems for the conversion of carbon dioxide to bicarbonates and vice-versa. It was the first zinc metalloenzyme to be discovered in living systems (Smith and Ferry, 2000). Zinc ion complex facilitates carbon dioxide hydration activity. In most of the organisms, CAs are required for rapid processes, particularly transport processes. For example, it is required for the removal of CO_2_ from lungs and for synthesis of eye secretions. CAs maintain optimum level of CO_2_ and ${\text{HCO}}_{3}^{-}$ in the body as they are utilized as substrate for many enzymatic reactions. It maintains acid—base balance in blood and helps in maintaining its physiological pH and also actively participates in ion transport and respiration. Mutations in CA genes can lead to osteoporosis and mental retardation. Carbonic anhydrase II (hCAII) is present in relatively high concentrations in red blood cells (Berg et al., 2002).

Initially the presence of the enzyme was found in the animal kingdom but later on CAs showed their signatures in all three living domains (Kaur et al., 2012; Di Fiore et al., 2015). This enzyme is either found intracellularly in cytoplasm or secreted outside (extracellular) associated with the periplasm and an essential component for survival of nearly all life forms. CAs are one of the fastest among the enzymes known having k_cat_ in the order of 10^6^ s^−1^ and k_cat_/K_m_ in the order of 10^8^M^−1^s^−1^. An “anhydrase” is defined as an enzyme that catalyzes the removal of a water molecule from a compound, and so it is this “reverse” reaction that gives carbonic anhydrase its name, because it removes a water molecule from carbonic acid (Smith et al., 1999). In plants, β—CAs have a role in photosynthesis in chloroplasts by raising the concentration of CO_2_ to enhance the carboxylation rate of ribulose 1, 5-bisphosphate carboxylase (RuBisCO) (Smith and Ferry, 2000). It functions in three modes: conversion of CO_2_ to bicarbonate (to be utilized by RuBisCO in C_4_ plants), conversion of bicarbonate into CO_2_ [for fixation by phosphoenol pyruvate carboxylase (PEPC)] and also aids in facilitated diffusion by rapid equilibration between CO_2_ and ${\text{HCO}}_{3}^{-}$. It also provides bicarbonate, which is required for the metabolism in plants (Monti et al., 2013). Recently β–CA has been shown to play a role in the perception of salicylic acid in *Arabidopsi thaliana*, suggesting its requirement in defense response (Medina-Puche et al., 2017). Besides higher organisms, CAs are also required in lower organisms. In some of the heterotrophs such as *Propionibacterium*, CAs help in CO_2_ reduction during glycerol fermentation that results in the formation of oxaloacetate (Wood et al., 1941). In cyanobacteria and microalgae, CAs are involved in the CO_2_ concentrating mechanism (CCM), which helps the cells to photosynthesize in the absence of inorganic carbon and also due to decline in levels of CO_2_ in their surrounding environment (Badger and Price, 2003). CCM helps in maintenance of CO_2_ levels around the RuBisCo active centers thereby improving the efficiency of Calvin cycle. CAs have also been reported from facultative anaerobes such as *Rhodospirillum rubrum* (Gill et al., 1984). CAs supply the cellular transporters with ${\text{HCO}}_{3}^{-}$ by converting CO_2_ penetrating the cells into ${\text{HCO}}_{3}^{-}$. Extracellular CA in alkaliphilic cyanobacteria plays a role in their survival in high alkaline conditions in alkaline soda lakes (Soltes-Rak et al., 1997; So et al., 1998; Kupriyanova et al., 2007, 2011). In *Microcoleus* and *Rhabdoderma*, CA doesn't allow CO_2_ to leak out from the cell by converting it into bicarbonate, thus preserving the intracellular C_i_ pool for photoautotrophic assimilation. In *Rhabdoderma*, out of two CAs present, one is bound to photosystem II (PSII) of thylakoid membranes. It participates in the light photosynthetic reactions, regulating operation of the water oxidizing complex via its protection against excess of protons, similar to luminal CA of microalgae and higher plants (Shutova et al., 2008). CA in cyanobacterial thylakoid membranes supplies CO_2_ for photosynthesis in cyanobacteria. CA helps in the formation of oxaloacetic acid from carboxylase and phosphoenolpyruvate (PEP) carboxylase by providing bicarbonate which is utilized by pyruvate (Norici et al., 2002). This oxaloacetate is used for the synthesis of aspartate family of amino acids. Lysine production increases in elevated CO_2_ conditions owing to the action CA and PEP carboxylase (Puri and Satyanarayana, 2010). CA in carboxysomal shell of chemolithoautotrophic cyanobacterium *Halothiobacillus neapolitanus* (CsoS3) supplies the active sites of RuBisCO with high concentrations of CO_2_ necessary for RuBisCO activity and efficient carbon fixation (So et al., 2004). β–CA present in *Escherichia coli* is a major player in the cyanate degradation pathway of the organism. This type of CA has also been shown to play a role in pathogenesis of some bacteria such as *Salmonella typhimurium* (Valdivia and Falkow, 1997). In methanogens, CA plays an active role in acetate metabolism by converting the excess carbon dioxide produced into bicarbonates. It also helps in the conversion of acetate into methane. γ–class homologs of the CamH subclass are found in mitochondria, where it might have a role in the carbon transport system to increase the efficiency of photosynthetic carbon dioxide fixation (Tripp and Ferry, 2000). Hence CA and its classes play an important role in metabolic functions of all life forms. Life without CA is virtually out of question. CA levels are also correlated with aging (Cabiscol and Levine, 1995). A variety of diseases have been associated with such oxidative damage, which includes Parkinson's disease, diabetes, rheumatoid arthritis and alzheimer's disease. Classes of CA have been briefly described in the next section.

### Types of CA and mechanism of action

There are basically six types of CAs discovered till now, namely α, β, γ, δ, ζ, and η. They don't have any specific sequence similarity, hence representing a classical case of convergent evolution (Lane and Morel, 2000; Smith and Ferry, 2000; Lapointe et al., 2008; Del Prete et al., 2014). Characteristic features of these classes are briefly described in Table 1.

**Table 1:** Characteristic features of basic six classes of carbonic anhydrases.

**Characteristics**	**α CA**	**β CA**	**γ CA**	**δ CA**	**ζ CA**	**η CA**
**Occurrence**	Predominantly in animal kingdom also found in protozoa, algae, green plants, and in some archaea, bacteria and fungi	Reported in all three domains of life	Eubacteria and Archaea *Methanosarcina thermophila*	*Thalassiosira weissfogii*	*Thalassiosira weissfogii* (marine diatom)	*Plasmodium falciparum*
**Molecular weight**	monomeric (Mw: 26-37 kDa)	dimer, can form various olgomeric structures 45 to 200 kDa	homotrimer 60 kDa (native)	monomer27 kDa	69 kDa	-
**Active site amino acids**	His – 94, His – 96, His – 119	Cys 38, His 96 and Cys 99	His 81, His 122 and His 117	-	-	His – 94, His – 96, His – 118
**Function**	pH homeostasis, secretion of ${\text{HCO}}_{3}^{-}$, ion exchange	cyanate degradation (*E.coli*), CO_2_ fixation (cyanobacteria), Overexpressed during pathogenesis of some pathogens (*Salmonella*)	acetate metabolism and conversion of acetate to methane (methanogens)	-	-	Overexpressed during pathogenesis
**Other features**	esterase activity, N – terminal signal peptide for periplasmic localisation or extracellular secretion	Basic component of Carbon Concentrating Mechanism (CCM)	Oldest form of CA	-	cambialistic	

Despite their structural differences, CAs have similar action mechanism. This mechanism has been widely studied in α–CAs. It is a two–step ping-pong reaction that catalyzes the reversible hydration/dehydration of CO_2_ into bicarbonate and a proton (Silverman, 1982). A histidine residue present near the water molecule accepts a proton (H^+^) which gets released from the water molecule. It leaves only hydroxide ion attached to zinc ion. The active site has specific pockets for binding CO_2_, where it gets bound to the hydroxide ion. Further, nucleophilic attack on the carbonyl group by the zinc-bound hydroxide takes place that results in ${\text{HCO}}_{3}^{-}$ formation. The enzyme is then regenerated and the bicarbonate ion is released (Lindskog and Coleman, 1973; Silverman and Lindskog, 1988).

(1) $${\text{EZnOH}}^{-}+{\text{CO}}_{2}+{\text{H}}_{2}\text{O}↔{\text{EZnH}}_{2}\text{O}+{\text{HCO}}_{3}^{-}$$

(2a) $${\text{EZnH}}_{2}\text{O}+\text{PSR}↔{\text{EZnOH}}^{-}+\text{PSR}-{\text{H}}^{+}$$

(2b) $$\text{PSR}-{\text{H}}^{+}+\text{B}↔\text{PSR}+\text{B}-{\text{H}}^{+}$$

### CA specific inhibitors and their biological relevance

CA abnormality leads to several diseases such as edema, glaucoma, hypertension, epilepsy, and cancer (Supuran, 2008). Pharmaceuticals that suppress the activity of carbonic anhydrase are classified as carbonic anhydrase inhibitors. Their clinical use has been established as diuretics, anti-glaucoma agents and antiepileptics in the management of gastric and duodenal ulcers, mountain sickness, neurological disorders, or osteoporosis (Supuran et al., 2003). Among human CA isoforms, CAIX and CAXII have already been identified as potent drug targets and molecular markers for the treatment of various types of cancer and tumors. Both show upregulated expression in cancerous cells as compared to normal cells. The two isoforms are required for maintaining intracellular pH of tumor cells. The inhibition of these two CAs is, therefore, important for cancer treatment. Several workers and companies have developed several ureido and sulfonamide based pharmaceuticals for cancer treatment which inhibit CAIX and CAXII (Cabiscol and Levine, 1995; Lomelino and McKenna, 2016).

### Sulfonamide, sulfamate, and sulfamide inhibitors

Sulfonamides (R–NH_2_SO_2_) constitute an important group of classical drugs which have been known for their pharmaceutical and CA inhibitory properties. The most effective are the heterocyclic sulfonamides (acetazolamide, methazolamide, ethoxzolamide). Acetazolamide is one of the well**-**known inhibitors of carbonic anhydrase and has rendered its effectiveness for the *in vivo* inhibition of intracellular CAs (Teicher et al., 1992). It is used in the treatment of glaucoma, epilepsy (rarely), idiopathic intracranial hypertension, and altitude sickness. Similarly other sulfanamide inhibitors such as dorzolamide and brinzolamide are used in the treatment of glaucoma as well. Near about 20 CA inhibitors have been granted FDA approval and they are being put to wide clinical usage (More et al., 1946; Sugrue, 2000; Frost and McKenna, 2013; Wulf and Matuszewski, 2013; Supuran, 2016a). Sulfamic acid (NH_3_SO_3_) and sulfamide (NH_2_SO_2_NH_2_) are the simplest CA inhibitors containing the –NH_2_SO_2_ moiety. However, the sulfonamides are less effective against some of the β–CAs like Cab and also some of the γ–CAs (Zimmerman et al., 2004) and fungal MG-CA where they inhibit in millimolar/micromolar concentrations. Sulfamic acid and sulfamide are the weakest with inhibition constants (*K*_*i*_) in the millimolar range. Interestingly, a completely different inhibition profile was observed against Zn-Cam and MG-CA, where sulfamic acid and sulfananilamide were shown to be more potent than sulfonamide inhibitors. Over the years various other inhibitors of CAs (benzenesulfonamides, arylbenzenesulfonamides, tetrafluorobenzenesulfonamides, 4-aminoethylbenzenesulfonamide) have been synthesized/discovered and their potential inhibitory properties have been evaluated (Pastoreková and Pastorek, 2004; Pala et al., 2014). Two CAs (LpCA1 and LpCA2) from *Legionella pneumophila*, a pathogenic bacterium, also get inhibited by sulfanamide inhibitors (sulfonylated aromatic sulphonamides, acetazolamide, ethoxzolamide, methazolamide, and dichlorophenamide). They can serve as a promising drug target for this pathogen (Supuran, 2016a). MGM Institute of Health Sciences has developed cerium oxide nanoparticles which are plant based that exhibit CA inhibitory and antioxidant properties (Lomelino and McKenna, 2016). An electrochemical enzyme inhibition biosensor, based on CA entrapped in a carbon paste electrode using carbon black nanoparticles and solid paraffin, was developed that measures sulfanilamide inhibition of CA (Bourais et al., 2017). This biosensor can even detect the sub-micromolar levels of sulfanilamide and the detection limit was 0.4μm (Bourais et al., 2017).

### Novel inhibitors

Coumarins, phenols, polyamines, fullerenes, boronic acid and their substituted derivatives have been effective against animal CAs (Innocenti et al., 2009b; Carta et al., 2012; Supuran, 2013). Their efficacy against microbial CAs, however, must be investigated. Carta et al. (2012) recently introduced dithiocarbamates (DTCs) as a new class of Zn-binding CA inhibitors, which can interact with the nearby amino acid residues for effective binding. Indeed these promising inhibitors have been effective even in sub-nanomolar concentrations. Nitroimidazoles decrease the pH of the hypoxic cancer cells, thereby helping in the uptake of chemotherapeutic agents. Scientists are also developing CA inhibitory antibodies for cancer treatment (Lomelino and McKenna, 2016). Incorporation of sugar moieties into sulfanilamide has resulted in sharp improvement in their solubility and effectiveness. The inhibition constants for ribose and galactose sulfanilamides against bsCA1 were reduced to 8.9 and 9.2 nM as compared to 2,500 nM for the unsubstituted sulfanilamide. In contrast to other sulfonamides and sulfanilamides, glycosylsulfanilamides have a good balance between its hydro- and liposolubility, therefore, it can easily penetrate through membranes to affect the growth of microorganisms (Supuran, 2015). Mete et al. (2016) synthesized a series of new thienyl-substituted pyrazoline benzenesulfonamides and showed their effective inhibition against hCAI and hCAII.

### Thermo-alkalistable carbonic anhydrases

Advances in recombinant DNA technology have enabled us to modify/change a protein's structure and function as per the demand. As already stated, utilization of CAs in biomineralization of CO_2_ requires the enzyme to be alkalistable as the mineralization is favored at alkaline pH (Farrell, 2011) and the temperature of flue gas is too high (around 140°C), which is cooled to about 60°C for post-combustion CO_2_ capture. Hence, the enzymes needed for carbon sequestration must be thermostable. Several efforts have been made to modify the mesophilic CAs in order to make them thermo-alkali-stable. CO_2_ Solutions Inc. has developed a thermally optimized CA by genetic engineering which is stable at 90°C for 24 h (CO_2_ Solutions Inc., 2012). Fisher et al. (2012) modified the surface amino acid residues of hCAII (which are far away from active site) to increase the thermostability by 6°C. Some of the amino acid residues present at the surface (Tyr7, Leu224, Leu100, Leu240, Asn67, and Asn62) were replaced by Phe, Ser, His, Pro, Gln, and Leu, respectively. Directed evolution technique was utilized in order to develop a thermostable CA from β–CA from *Desulfovibrio vulgaris*. It can even retain activity at 107°C. The enzyme was stable in the presence of primary flue gas contaminats such as NOx and SOx as well as in the presence of 4.2 M concentration of N-methyl-diethanolamine (MDEA) at 50°C for about 14 weeks. The enzyme retains 40% residual activity at alkaline pH (11.8). This highly stable CA has been efficiently used for biomineralization of CO_2_ at high temperature (87°C) (Alvizo et al., 2014). A whole cell bacterial catalyst was generated from the CA of *Neisseria gonorrhoeae* (*ng*CA) by engineering it in such a way that it is secreted in the periplasm of *E*. *coli*. This whole cell catalyst was also found stable at low pH. It might be possible to sequester CO_2_ more efficiently using whole cell enzyme systems even at a pH below the pKa of HCO^3−^ or ${\text{CO}}_{3}^{2-}$, thereby reducing the cost of maintaining it at elevated pH (Jo et al., 2013). The modified CA thus generated by these strategies can aid in efficiently capturing CO_2_, but adds to the cost. It is worthwhile to search for enzymes that are stable at two/more extreme conditions. The use of thermo-alkali-stable CA from polyextrmophiles would simplify the process. Jo et al. (2016) engineered the de-novo sulfide bond of ngCA which resulted in enhancement in both kinetic and thermodynamic stabilities. The major reason for this enhancement is the loss of conformational entropy of the unfolded state, thereby increasing rigidity.

Recent advances in technology have enabled biologists to reach the extreme maxima of earth, sea and sky for exploring their diversity. Extremophiles have been isolated from a wide array of extreme environments like deep sea vents, hot springs, upper troposphere and stratosphere, outer space and others (Wilson and Brimble, 2009). There are also reports on the isolation of polyextremophiles from mines and industries (Onstott et al., 2003; Bhojiya and Joshi, 2012). Their characterization and applications have added new dimension to applied biology. Polyextremophiles are known to produce a variety of useful products (Coker, 2016). Metagenomics and data mining studies have revealed the presence of α, β, γ CA genes in microbes from stressed environments. β–CA from *Methanobacterium thermoautotrophicum* (CabCA) and γ–CA from *Methanosarcina thermophila* (CamCA) were the first known CAs from extremophiles (Alber and Ferry, 1994; Smith and Ferry, 2000). Two novel and highly thermo-alkali-stable α–CAs (SazCA and Ssp CA) have been discovered from thermophilic archaea *Sulfurihydrogenibium azorense* and *Sulfurihydrogenibium yellowstonense*. YO3AOP1, respectively. The former being one of the fastest known CAs till date (k_cat_/ K_m_ value of 3.5 × 10^8^M^−1^ s^−1^) (De Luca et al., 2013). SSp CA is even active after 3 h incubation at 70°C. SazCA is also highly thermostable having 53 days and 8 days of half-life at 40° and 70°C, respectively. It retains carbon dioxide hydration activity even after incubation at 80 and 90°C for several hours (Russo et al., 2013). Both these enzymes are alkalistable (active at pH 9.6) and stable in the presence of flue gas contaminants such as NO^2−^, NO^3−^, and ${\text{SO}}_{4}^{2-}$ (Vullo et al., 2012; De Luca et al., 2013). Two highly thermophilic bacteria isolated from hydrothermal vent ecosystems *Persephonella marina* EX-H1 (PMCA) and *Thermovibrio ammonificans* produced highly thermostable α–CA. The CA of *T. ammonificans* (TaCA) was more stable than SazCA and SspCA. This enzyme (taCA) showed thermo-stimulating properties (activity of taCA was elevated after the high temperature incubation (Jo et al., 2014). Faridi and Satyanarayana (2016a) reported a moderately thermostable and highly alkalistable α–CA(BhCA) from polyextremophilic bacterium *Bacillus halodurans*. It has a unique property of sulfate stimulation (its activity enhanced in the presence of sulfate ions). This property can be exploited for CCS as the flue gas contains SOx as one of the contaminants. BhCA did not get denatured in the presence of EDTA (Faridi and Satyanarayana, 2016b). Bose and Satyanarayana (2016) reported a moderately thermostable and alkalistable γ–CA from *Aeribacillus pallidus*, a polyextremophilic bacterium. Both ApCA and BhCA were stable in presence presence of bicarbonate (0.1M). *Psychrobacter* sp. SHUES1 isolated from frozen alkaline samples from Shanghai (China) produced carbonic anhydrase and urease which are important in microbially induced carbonate precipitation (MICP; Li et al., 2016). List of some of the thermo-alkali-stable CAs which have been characterized in the last few years have been highlighted in Table 2 with their characteristics.

**Table 2:** Thermostable CAs and their characteristic features.

**Sl. No**.	**Enzyme**	**Organism**	**Class**	**Characteristic features**	**References**
1.	SspCA	*Sulfurihydrogenibium yellowstonense*. YO3AOP1	α	Dimer, Stable at high temperatures (70°C for 3 h) Optimum working condition–95°C, pH 9.6	Capasso et al., 2012
2.	Saz CA	*Sulfurihydrogenibium azorense*	α	Dimer, Half-life at 70°C is 8 days and 53 days at 50°C. Alkalistable (pH 9.6)	De Luca et al., 2013; Russo et al., 2013
3.	TaCA	*Thermovibrio ammonificans*	α	77 days of half-life at 60°C, tetramer	Jo et al., 2014
4.	PmCA	*Persephonella marina* EX-H1	α	88 days of half-life at 100°C, Dimer	Jo et al., 2014; Kanth et al., 2014
5.	Cab CA	*Methanobacterium thermoautotrophicum*	β	Tetramer Optimal CO_2_ hydration activity at 75°C	Smith and Ferry, 2000
6.	MtCam (aerobically purified)	*Methanosarcina thermophila*	γ	Optimally active at 55°C and displayed 15 min of stability at 75°C, Activity doubles when zinc is replaced by cobalt	Alber and Ferry, 1994
7.	MtCam (anaerobicaly purified)	*Methanosarcina thermophila*	γ	NA	Tripp et al., 2004
8.	MtCam (expressed in *M. acetivorans*)	*Methanosarcina thermophila*	γ	Fe^2+^ present at active site enhances its activity	MacAuley et al., 2009
9.	BhCA	*Bacillus halodurans*	α	Thermo-alkali-table (pH 6–11), gets stimulated in the presence of SOx, stable with EDTA	Faridi and Satyanarayana, 2016a
10.	ApCA	*Aeribacillus pallidus*	γ	Thermo-alkali-stable (pH 8–11), stable in the presence of SOx and NOx	Bose and Satyanarayana, 2016

Crystal structures of three of the most thermo-alkali-stable CAs were solved and their analysis revealed the reasons of their higher thermostabilities. As per the crystallographic structure, SspCA was found to have increased structural compactness. It contained a large number of charged residues on the protein surface with a greater number of ionic networks. These might be the key factors involved in the higher thermostability of this enzyme with respect to its mesophilic homologs. It has a fold which is characterized by a 10—stranded centrally placed β–sheet, which is surrounded by several helices and β–strands. A deep conical cavity that extends from the center to the protein surface harbors the active site. Several polar and hydrophobic interactions play active role in stabilization of SspCA. SazCA has a similar structure to that of SSpCA with minor differences. SspCA has Glu2 and Gln207 residues which are substituted with His2 and His207 in SazCA. This substitution is responsible for higher SazCA catalytic activity. The crystallographic structures of both SazCA and SspCA confirmed the dimeric nature of the enzymes (Di Fiore et al., 2013; De Simone et al., 2015). Crystallographic structure analysis of TaCA revealed it to be similar to the structure of other previously known bacterial homologs (Huang et al., 1998; Di Fiore et al., 2013; De Simone et al., 2015), but having entirely novel oligomeric pattern. Indeed TaCA forms a tetramer that comprises two dimers, which are structurally similar to that of SazCA and SspCA. The two dimers are joined together by two intermolecular disulfide bridges and by inter-subunit ionic interactions (James et al., 2014). This tetrameric state may be a possible reason for the enhanced thermostability of TaCA. Tahirov et al. (1998) recorded that thermostable enzymes have higher degree of oligomerization than mesophilic enzymes. It is also worthwhile to mention that the two conserved cysteine residues, Cys202 and Cys47, are reduced partially in SspCA and SazCA due to insufficiently oxidative expression conditions. It is suggested that the presence of two cysteine residues in TaCA leads to its increased stability (James et al., 2014). These structural analyses of thermostable CAs not only provide structural insights of the enzymes but also aid in their modification, so that they can be efficiently utilized for biomineralization.

### Utilization of thermo-alkali-stable CAs in biomineralization

Several strategies are being developed for utilization of thermo-alkali-stable CAs in mitigating global warming. CO_2_ Solutions Company has developed a CA based reactor for capturing CO_2_ from different CO_2_ intensive industries (http://www.co_2solutions.com/en/the-process). This process requires immobilized CA. Utilization of thermo-alkali-stable CAs for carbon capture requires a series of bioreactors which will have direct supply of flue gas. It is directly supplied to a bioreactor containing the immobilized enzyme. This enzyme in aqueous condition will hydrate CO_2_ present in flue gas and releases ${\text{HCO}}_{3}^{-}$ and H^+^. Alvizo et al. (2014) developed a process which utilizes a highly thermostable β–CA engineered from the β–CA of *Desulfovibrio vulgaris*. In this process, the set up consists of absorber column where carbon dioxide gets absorbed into MDEA; CA present in the column generates proton and bicarbonate. The residual flue gas is released and the rest (bicarbonate+amine+CA) is transferred to the next column maintained at 87°C, where CO_2_ is again regenerated, accompanied by regeneration of the solvent. Pure CO_2_ is later reutilized and the solvent returns back to the initial absorber column for the next cycle. The rate of CO_2_ absorption increases by about 25-fold in the catalyzed reaction as compared to the non-catalyzed reaction. Normal conditions for steady-state experiments were 2 l per minute of solvent, 180 l per minute of flue gas at 25°C and absorber and desorber temperature at 87°C. All enzyme–solvent mixtures were made using normal tap water without any further treatment (Alvizo et al., 2014). CAs from *Caminibacter mediatlanticus* (CmCA) and *Sulfurihydrogenibium yellowstonense* YO3AOP1 (SspCA), both belonging to α–class, have been used to capture CO_2_ from flue gas (Daigle and Fradette, 2014; Rossi, 2014). SspCA was characterized as prospective biocatalyst for CO_2_ capture as it has regenerative absorption ability in alkaline conditions. Its prolonged half life (53 days at 40°C and 8 days at 70°C) making it one of the most suitable candidates for CCS. Ssp CA was, therefore, tested for its biomimetic carbon sequestration (Russo et al., 2013). Ssp CA immobilized in polyurethane foam (PU-SspCA) has been used in designing a bioreactor to mimic CO_2_ capture process in industries (Migliardini et al., 2014). The immobilized CA (PU-SspCA) showed exceptional thermostability for very long duration even at 70°C and was highly stable at 100°C even after 48 h (Capasso et al., 2012). Heat stable carbonic anhydrases from *M. thermophila* and *Caminibacter* sp. have been used in bioreactors for efficient CO_2_ removal from flue gases. *Pyrococcus horikoshii* and *M. thermophila* secreted γ–CA which was used in developing γ–CA nanoassemblies. These assemblies were developed by joining the single entitites to make multiple linked interactions with the surface of the reactor. Biotinylation sites were created by specifically mutating some of the residues to cysteines. Firm nanostructures were thus created by cross-linking biotinylated-γ–CAs with streptavidin tetramers (Salemme and Weber, 2014).

**Figure 1:**
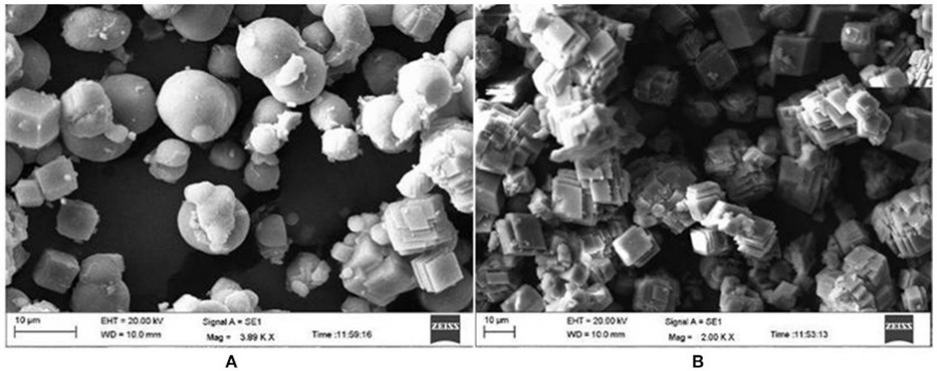
Scanning electron microscopic picture of calcium carbonate crystals in the presence and absence of CA (control). **(A)** Spherical vaterite crystals were formed in the control. **(B)** Rhombohedral and well faceted calcite crystals were formed in the presence of ApCA (adopted from Bose and Satyanarayana, 2016).

There are basically three different crystal phases for calcium carbonate (CaCO_3_), viz. calcite (rhombic), aragonite (needle like) and vaterite (spherical)]. Calcite phase is thermodynamically the most stable phase, while the vaterite is the metastable phase which is more soluble (Favre et al., 2009). When PMCA and TaCA (both α–CAs) were utilized for biomineralization; it resulted in the formation of stable calcite (Jo et al., 2014). α–CA from *Dunaliella* sp. produced 8.9 mg of calcite per 100 μg (172 U/mg) of enzyme in presence of 10 mM Ca^2+^ (Kanth et al., 2012). Another thermo-alkali-stable γ–CA (ApCA) from polyextremophilic bacterium *A. pallidus* was used for biomineralization and well faceted rhombohedral calcite crystals were observed when viewed under scanning electron microscopy (SEM; Figure 1). The partially purified enzyme caused precipitation of 42.5 mg ${\text{CO}}_{3}^{2-}$ mg^−1^ protein (Bose and Satyanarayana, 2016). Optimization of the process parameters led to an increase in carbonate precipitation up to 200 mg ${\text{CO}}_{3}^{2-}$ mg^−1^ protein (Bose and Satyanarayana, 2017).

**Figure 2:**
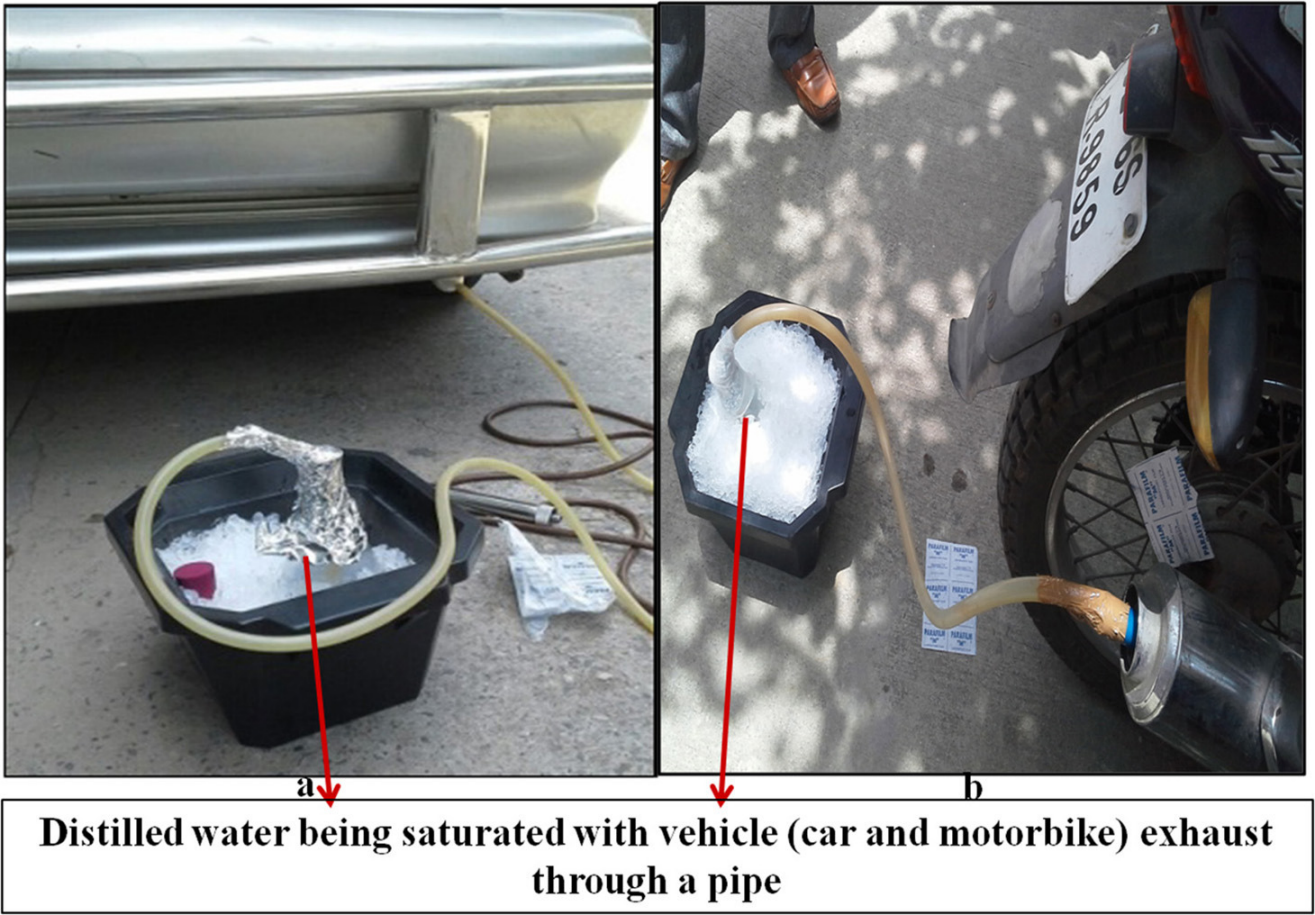
Exhaust fumes from the petrol driven car and motorcycle were collected by connecting one end of a plastic pipe to the exhaust of the car and the other to saturate distilled water (DW) kept in ice bath for an hour which served as a source of CO_2_ (adopted from Faridi and Satyanarayana, 2016a).

**Figure 3:**
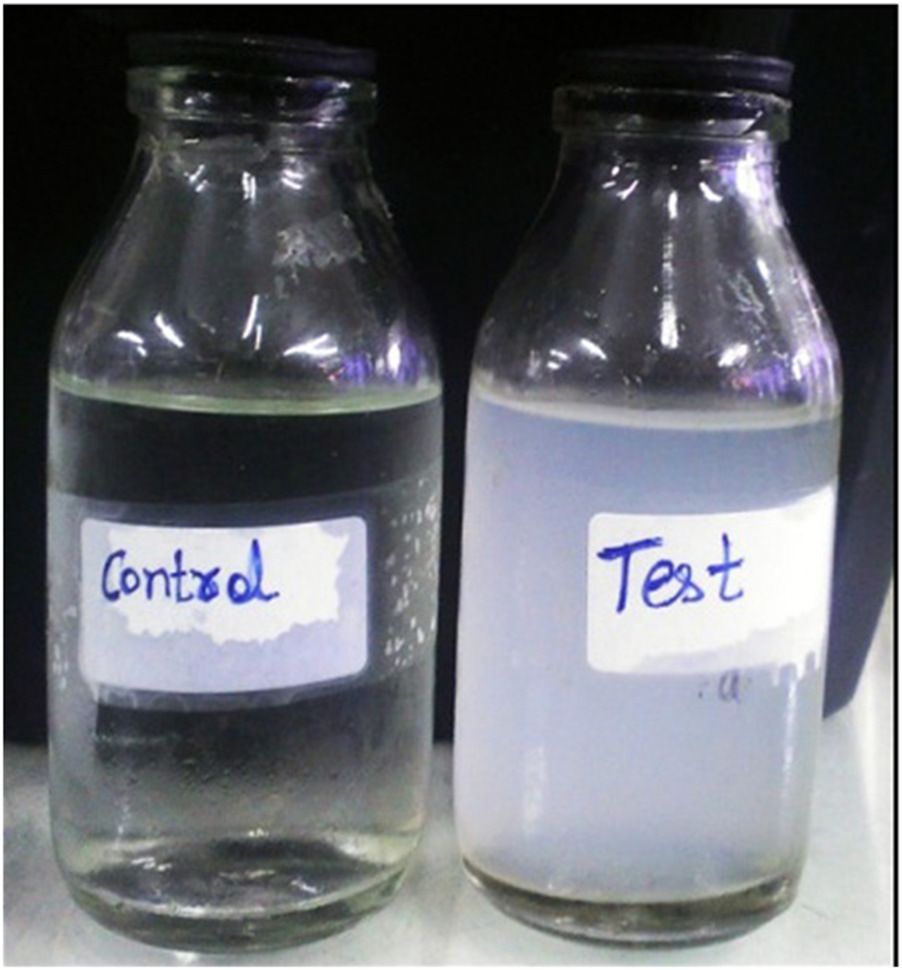
Mineralization of vehicular exhaust gas CO_2_ using BhCA in the presence of Ca^2+^. Test reaction containing CA showed efficient mineralization of CO_2_ as compared to the control (adopted from Faridi and Satyanarayana, 2016a).

When a highly alkalistable and moderately thermostable α–CA (BhCA) from *Bacillus halodurans* was used for biomineralization, well defined calcite crystals were observed (Faridi and Satyanarayana, 2016a). ApCA and BhCA were also utilized for testing their efficacy in CO_2_ sequestration from flue gas using vehicular exhaust (Figure 2). Vehicular exhaust has a comparable composition as that of flue gas emitted from the thermal power plants (~71% N_2_, 14% CO_2_, 1–2% hydrocarbons, carbon monoxide, NO_x_, and minor amount of SO_2_; Perry and Gee, 1995). Both the enzymes were useful in sequestering CO_2_ from vehicular exhaust, hence making them efficient candidates for biomineralization (Faridi and Satyanarayana, 2016b; Figure 3). Biomineralization process for ApCA and BhCA was performed at 37°C in a 75 mL total reaction volume. CA (0.05 mg) was dispersed in 15 mL Tris buffer containing 0.9 g of CaCl_2_.2H_2_O in Tris (2.52 g) buffer according to a protocol used by Mirjafari et al. (2007). To initiate the mineralization process, 60 mL of CO_2_ solution was added to the enzyme mix. The reaction was performed for 10 min at 37°C. In an interesting study, the effect of CA from *Bacillus mucilaginosus* on carbonate formation and wollastonite dissolution were explored under variable CO_2_ conditions. Real time PCR was used to analyze the correlation between CA gene expression, sufficiency or deficiency of calcium and CO_2_ concentration. The findings reaffirmed the belief that CO_2_ concentration is not related to effects of CA. Further, they also showed that microbial CA has some role in silicate weathering (Xiao et al., 2014). Some of the other alkalistable CAs have also shown efficacy in carbon sequestration. Another α–CA from *Serratia* sp., which was alkalistable in nature, was successful in generating calcite crystals (Srivastava et al., 2015). Some of the mesophilic CAs from some other microorganisms such as *Pseudomonas fragi* (27.33 mg ${\text{CO}}_{3}^{2-}$/mg of protein), *Bacillus pumillus* (25.43 ${\text{CO}}_{3}^{2-}$/mg of protein), *M. lylae* (24.02 ${\text{CO}}_{3}^{2-}$/HCO^3−^/mg of protein) (Prabhu et al., 2011) and *Citrobacter freundii* SW3 (Ramanan et al., 2009) [225 mg CO3^2−^/mg of protein] also produced calcite crystals. *Rhodobacter sphaeroides* was genetically modified to harbor a surface displayed CA with inducible expression of phosphoenolpyruvate carboxylase led to very high CO_2_ reduction efficiency and the production of several organic compounds (carotenoids, polyhydroxybutyrate, malic acid, succinic acid; Park et al., 2017).

### Production and over expression of carbonic anhydrase genes by molecular approaches

One of the key factors that determine the cost effectiveness of any biotechnological application is the availability and cost of the biocatalysts. Microorganisms are well known to produce CAs for use in CCS technologies. Researchers have also displayed the ability of many prokaryotic species such as planktonic *Bacillus mojavensis* cells for microbially enhanced CCS by mineral trapping and solubility trapping (Mitchell et al., 2010). The production levels of CAs are, however, quite low for industrial use. Hence the production parameters must be optimized. Statistical approaches such as Response surface methodology (RSM) and two factorial designs are some of the well-known methods for optimizing production of biocatalysts (Kumar and Satyanarayana, 2014). The production of carbonic anhydrase is growth associated; hence, optimization of growth parameters leads to high enzyme titres. Optimization of growth conditions by mono-factor tests with blank control and orthogonal design methodology resulted in 7-fold increase in CA titres in *B. mucilaginosus* K02 (Zhang et al., 2011). Bose and Satyanarayana (2016) reported about 4-fold increment in enzyme titres on optimization of growth parameters. In both the studies, the CA production was found to be synchronous to that of the growth of the bacterium. The CA production by the wild type strain becomes too cumbersome. The production of CA from thermo-alkaliphilic microorganisms is difficult as it is not easy to provide accurate conditions [anaerobic, high temperature, pH (high/low) etc.] for their growth and enzyme production. Many archaeal life forms producing bioactive compounds need specialized growth conditions (Alber and Ferry, 1994; Mesbah and Wiegel, 2012). The advent of genome mining, metagenomics and bioinformatics approaches have enabled to use molecular biological tools for cloning and over expression of industrially relevant enzymes in prokaryotic (*E. coli, Bacillus subtilis*) and eukaryotic (*Pichia pastoris*) hosts. Downstream processing becomes easy as the enzymes have various protein tags (e.g., His, GST) for easy purification of the recombinant proteins (Swartz, 2001). Since late 1990s, the prokaryotic CA genes from different strata have been cloned and the protein was over expressed in *E. coli* for analytical and application purposes. The first CA gene cloned from prokaryotes was α–CA (NCA) from *N. gonorrhoeae*, which encoded a protein of 28 kDa (Chirică et al., 1997). This enzyme was also expressed heterologously in *E. coli* as a periplasmic protein in order to use it as a whole cell biocatalyst (Jo et al., 2013). The yield of ~106 mg L^−1^ with a specific activity of 3090 U mg^−1^ in the pure protein was achieved (Kim et al., 2012).

Since then many other microbial CAs have been cloned and expressed in prokaryotic hosts. Many researchers have already cloned and over expressed all the CA-encoding genes from *E. coli* (*can, cynT, cynT2, caiE, pay*, and *yrdA*) and studied their expression profiles in response to different growth conditions (Fujita et al., 1994; Cronk et al., 2001; Merlin et al., 2003). CA genes from thermophilic bacteria such as *Thermovibrio ammonificans, M. thermobacterium, M. thermophila, Sulfurihydrogenibium, B. halodurans* and others have been cloned and over expressed in *E*. *coli* to assess their efficacy in biomineralization. These CAs have been shown to be useful in sequestering CO_2_ from flue gas (Capasso et al., 2012; Faridi and Satyanarayana, 2016a). The role of CAs in virulence/pathogenesis have been studied. They play an active role in metabolism during pathogenesis in many bacterial and fungal pathogens such as *Vibrio cholerae, Helicobacter pylori, Candida albicans, Aspergillus fumigatus*, and others. List of CAs from some of the pathogens have been summarized in Table 3.

**Table 3:** Characteristics of CA from some pathogenic bacteria.

**Sl. No**.	**Organism**	**Type**	**Cloning Vector/Host**	**Molecular weight**	**Other Features**	**References**
1	*V. cholerae*	α	*E. coli* (pET15b)	26.4 kDa	K_m_ = 11.7 mM k_cat_ = 8.23 × 10^5^s^−1^ k_cat_/K_M_ = 7.0 × 10^7^ M^−1^ s ^−1^	Del Prete et al., 2012
2	*Brucella suis*	B	*E. coli* (pET15b)	25 kDa	k_cat_ = 1.1 × 10^6^, k_cat_/K_m_ = 8.9 × 10^7^ M^−1^ s^−1^	Joseph et al., 2010
3	*H. pylori*	α	*E. coli* (pACA1)	28.28 kDA	k_cat_ = 2.4 × 10^5^s^−1^ K_M_ = 17 mM kcat/K_M_ = 1.4 × 10^7^ M^−1^s^−1^ at pH 8.9 and 25°C	Chirica et al., 2001
4	*Salmonella typhimurium*	β (two stCA1 and stCA2)	*E. coli* (pGEX-4T2)	24.8 kDa (stCA1) 26.6 kDa (stCA2)	k_cat_ = 0.79 × 10^6^ s^−1^ and 1.0 × 10^6^ s-^−1^, k_cat_/K_m_ = 5.2 x10^7^M^−1^s^−1^ and 8.3 × 10^7^ M^−1^ s^−1^	Vullo et al., 2011
5	*Mycobacterium tuberculosis*	β (mtCA1)	*E. coli* pGEX-4T2	NA	k_cat_ = 3.9 × 10^5^ s^−1^ k_cat_/ K_m_ = 3.7 × 10^7^ M^−1^ s^−1^	Minakuchi et al., 2009
6	*Streptococcus pneumoniae*	β (PCA)	*E. coli*	NA	k_cat_ = 7.4 × 10^5^ s^−1^ k_cat_/K_m_ = 6.5 × 10^7^ M^−1^ s^−1^ at an optimum pH of 8.4	Burghout et al., 2011
7	*Plasmodium falciparum*	η (PfCA)	*E. coli*	NA	k_cat_ = 1.4 × 10^5^ s^−1^k_cat_/K_m_ = 5.4 × 10^6^ M^−1^s^−1^	Del Prete et al., 2014
8	*Haemophilus influenzae*	β (HICA)	*E. coli*	105.26 kDA (native molecular weight)	Tetramer, presence of a novel non-catalytic bicarbonate binding site, k_cat_, k_cat_/K_m_ is pH dependent	Cronk et al., 2006
9	*Candida albicans*	β (Nce103)		31.5 Da	k_cat_ = 8.0 × 10^5^ s^−1^k_cat_/K_m_ = 9.7 × 10^7^ M^−1^s^−1^	Innocenti et al., 2009a
10	*Malassezia globosa*	β (MG-CA)	*E. coli*	27 kDa	k_cat_ = 8.6 × 10^5^ s^−1^k_cat_/K_m_ = 6.9 × 10^7^ M^−1^s^−1^ K_i_ (acetazolamide) 76000 ± 450 nm	Hewitson et al., 2012
11	*Cryptococcus neoformans*	β (Can2)	*E. coli*	26 kDa	k_cat_ = 3.9 × 10^5^ s^−1^k_cat_/K_m_ 4.3 × 10^7^ M^−1^s^−1^K_i_ (acetazolamide) 10.5 nm (homodimer)	Mogensen et al., 2006; Innocenti et al., 2009a
12	*Aspergillus fumigatus*	4-βCAs (cafA-cafD)	*Saccharomyces cerevisiae*	–	All four CAs strongly expressed during pathogensis	Han et al., 2010; Tobal and Balieiro, 2014

One of the major problems with cloning of genes in heterologous hosts is obtaining the active protein in soluble form, as the recombinant proteins are aggregated as inclusion bodies, which need to be solubilised (Clark, 2001). Generally optimization of IPTG concentration, growth temperature (after induction) and incubation time brings the protein into the soluble form (Prasad et al., 2011). Techniques such as size-exclusion chromatography (Li et al., 2004), dialysis and dilution (Marston, 1986; Tsumoto et al., 2003; Umetsu et al., 2003), zeolite absorbing systems (Nara et al., 2009), reversed micelle systems (Sakono et al., 2004), microfluidic chips (Jahn et al., 2007) and the natural GroEL—GroES chaperone system (Zhi et al., 1992) could be used in soluble expression of recombinant proteins. Other problems include codon bias and lack of post translational machinery in *E. coli* (Gustafsson et al., 2004; Sahdev et al., 2008). The latter can be overcome by cloning the gene of interest in eukaryotic hosts such as *Saccharomyces cerevisiae* and *P. pastoris*. In this case, protein is expressed extracellularly, hence, we are saved from the energy intensive step of breaking the cells in disruptors or sonicators. Codon optimization also results in faster translation rates. α–CA from *Dunaliella* species was codon optimized and effectively expressed in *E. coli*, which was used for biosequestration of CO_2_ (Kanth et al., 2012). A synthetic α–CA (*HC-aCA*) from *Hahella chejuensis* was cloned and expressed (Ki et al., 2013). The codon-optimized carbonic anhydrase gene of thermophilic *Persephonella marina* EX-H1 (PMCA) was expressed and characterized. The removal of the signal peptide resulted in 5-fold enhancement of CA (Kanth et al., 2014).

### Role of metagenomics in CCS technologies

The advent of metagenomics has permitted discovery of numerous microorganisms from extreme environments, which could be of biotechnological interest. Metagenomics analysis has revealed the presence of CA encoding genes in the Indian Ocean viral metagenome (Williamson et al., 2012). Jones et al. (2012) showed the existence of RuBisCo and CA gene clusters in the *Acidithiobacillus*, an extremely acidophilic sulfur-oxidizing biofilm by community genome analysis. Microarray data of a metagenome of acid mine drainage also showed the presence of CA encoding genes along with the RuBisCo gene clusters (Guo et al., 2013). Three gene copies were identified which encoded CA in the metagenome of the marine ammonium oxidizing bacteria (van de Vossenberg et al., 2003). The metagenomes of the serpentinite-hosted Lost City hydrothermal field and Mid-Atlantic Ridge also indicated the presence of CA encoding genes. These metagenomes were found to be similar to the genome of *Thiomicrospira crunogena* XCL-2, an isolate from a basalt-hosted hydrothermal vent in the Pacific Ocean (Brazelton and Baross, 2010). Microorganisms exhibit great potential as bioindicators to detect leakages from CO_2_ storage projects and for that metagenomics becomes handy. Metagenomics and high throughput screen (HTS) methods can also be utilized for studying the effect of environmental changes on microbial communities at the CCS sites (Caporaso et al., 2012; Håvelsrud et al., 2012, 2013; Howe et al., 2014). Information about amplicons and metagenomes helps in establishing a CCS monitoring approach, which could even be useful in the detection of CO_2_ leaks (Noble et al., 2012). The use of HTS has already been found to be effective in evaluating the response of *in situ* bacterial populations to increased CO_2_, and matching community shifts to metabolic potential (Mu et al., 2014). The metagenomic approach along with appropriate bioinformatics tools makes the system competent. Metagenomics can thus be used as a biosensor for monitoring the CCS sites efficiently (Hicks et al., 2017).

### Utilization of immobilized CA in CCSU

Immobilization strategies are necessary so that the enzyme can be recycled number of times. Their stability can be enhanced by various immobilization techniques. Immobilization has long been used as an approach for increasing the stability of mesophilic enzymes. There are several reports of immobilization of mesophilic CAs. These CAs have been proved to be more efficient than the free enzymes for CO_2_ capture. CAs from *P. fragi, Bacilllus pumilus*, and *Micrococcus lylae* have been immobilized on chitosan and surfactant-modified silylated chitosan beads (Prabhu et al., 2009; Wanjari et al., 2011); these displayed enhanced CO_2_ hydration capacity. Chitosan is made of glucosamine and acetyl glucosamine units. The functional groups present on chitosan are amino and hydroxyl groups which are required for enzyme immobilization. The enzyme is adsorbed on the surface of chitosan beads. Immobilization of CAs also improved their thermal stabilities, for e.g., the immobilized CA retained at least 60% of the initial activity after 90 days at 50°C compared to about 30% for their free counterparts under the same conditions. The CAs also exhibit a high stability in the presence of inhibitors upon immobilization (Prabhu et al., 2009; Kanbar and Ozdemir, 2010). CaCO_3_ precipitation rate doubled in a period of 5 min when pure CA from *P. fragi* was immobilized by adsorption on chitosan beads in comparison with the free enzyme (Wanjari et al., 2012; Yadav et al., 2012). Immobilization of CAs on several other matrices such as ordered mesoporous aluminosilicate, octa(aminophenyl)silsesquioxane-functionalized Fe_3_O_4_/SiO_2_ nanoparticles, silica nanoparticles and by single/multiple attachments to polymers deposited on Fe_3_O_4_ particles was also attempted (Sharma et al., 2011; Rayalu et al., 2012). CA immobilized on ordered mesoporous aluminosilicate exhibited CO_2_ sequestration efficiency of 16.14 mg of CaCO_3_/mg CA as compared to that of free enzyme which sequesters 33.08 mg of CaCO_3_/mg CA. Immobilized CA even showed enhanced stability and retained 67% of initial activity even after six cycles (Yadav et al., 2011). Kinetics of the immobilized CAs (immobilized on ordered mesoporous aluminosilicate) were studied and compared with that of the free enzyme by Yadav et al. (2010). The *K*m, *V*max, and k_cat_ values of the immobilized enzyme were 0.158 mM, 2.307 μ mole min^−1^ml^−1^, and 1.9 s^−1^, and these for free CA were 0.876 mM, 0.936 μ mole min^−1^ml^−1^, and 2.3 s^−1^, respectively (Yadav et al., 2010). A high CO_2_ sequestration and improved stability have been achieved when CA was immobilized on core-shell CA-chitosan nanoparticles (SEN-CA; Rayalu et al., 2012). Ssp CA was immobilized on solid matrix made of silica particles (silanized Sipernat® 22 particles). Enzyme-carrier covalent bonding was adopted as immobilization technique and it was observed that the enzyme stability and activity increased on immobilization. Immobilization of this CA in polyutherane foam also exhibited enhanced stability. The immobilized CA (PU-SspCA) showed exceptional thermostability for very long duration even at 70°C. The CO_2_ absorption capacity of PU-SspCA was verified in a three phase trickled bed bioreactor which mimics the post-combustion processes in a thermal power plant. The three-phase reactor was filled with shredded foam with PU-SspCA. The gas mixture (CO_2_/N_2_) was fed from both the sides (i.e., concurrent and countercurrent). Increasing the flow rate of water and decreasing the CO_2_ flow rate also greatly improved CO_2_ capture in these reactors. SspCA showed good CO_2_ capture performance when the PU-SspCA-shredded foam was used in the bioreactor (Migliardini et al., 2014). Concerted efforts are needed to develop CA immobilization techniques that allow reuse of the enzyme 100–500 times with sustained activity. Immobilization can also lead to unwanted release of enzyme on surface of the reactor. In order to overcome this problem, some novel immobilization techniques have been developed using γ–CA from *P. horikoshii* and *M. thermophila* (Salemme and Weber, 2014). The immobilization techniques allowed the development of γ–CA nanoassemblies (Salemme and Weber, 2014). An immobilization sequence was added at amino- or carboxy-terminus which aided in proper and reversible immobilization of γ–CA to the functionalized surface. Gas-liquid membrane contractors are also emerging as potential bio reactors for using CAs for CO_2_ capture. Iliuta and Iliuta (2017) has demonstrated a novel, cost effective and environmentally friendly approach for CO_2_ capture by immobilizing CA in a hollow-fiber membrane bioreactor (*HFMB*) by multiscale steady-state model, under partially liquid-filled and gas-filled membrane pores conditions.

Nanoparticles are also widely used for immobilization of enzymes due to their unique size and physical properties. The immobilization of enzymes on nanoparticles (NPs) offers high surface area and may lead to reduction in protein unfolding, improvement in storage stability and performance (Laurent et al., 2008; Xu et al., 2014). Iron mangnetic nanoparticles (MNPs) are being synthesized with various surface modifications in order to use them for immobilizing protein/enzyme. The CA from *B. pumilus* TS1 was immobilized successfully on chitosan stabilized iron MNPs (Yadav et al., 2012). Silanization of iron MNPs is being widely used for surface modifications of iron MNPs. It is also very easy and cost effective technique, which can be carried out simply in aqueous or organic media at moderate temperatures (Xu et al., 2014). Faridi et al. (2017) showed that even after 22 cycles of reuse, recombinant α–CA (rBhCA) from *B. halodurans* TSLV1 immobilized on silanized iron MNPs (rBhCA-Si-MNPs) lost only 50% of activity. Nickel nanoparticles have been successfully used as a direct catalyst for CO_2_ hydration reaction for assessing their application in CO_2_ mineralization (Bhaduri et al., 2015).

### Other applications of carbonic anhydrases

Apart from their utility in CCSU, the CAs have some other applications too as discussed below:

### Carbonic anhydrase in formation of bioconcrete

Biomineralization by bacteria facilitates the development of bioconcrete, wherein calcium carbonate is formed by the metabolic activity of microorganisms, which involves a series of complex reactions directed mainly by urease and carbonic anhydrase enzymes (Castro et al., 2016). The activity of urease, CA, concentration of calcium and pH are very important in bioconcrete formation (Achal and Mukherjee, 2015).

### Artificial lungs

This equipment helps to overcome respiratory problems. The major drawback of this technique is the insufficient transfer of CO_2_ per square inch through the polymeric hollow fiber membranes (HFM). CAs can be utilized by immobilizing on HFM in order to increase the rate of CO_2_ transfer. CAs can, therefore, be used in developing small artificial lungs, which could be efficiently utilized within the human body (Kaar et al., 2007).

### Biosensors

HCA II based biosensors have been employed to check the toxic effects of zinc on marine life (Thompson and Jones, 1993; Rout and Das, 2003; Muyssen et al., 2006). Many researchers are attempting to develop biosensor variants for other transition metals (Thompson et al., 2000, 2002; Frederickson et al., 2006; Bozym et al., 2008; Wang et al., 2011; McCranor et al., 2012).

### Pharmalogical considerations

CAs can be incorporated into stimuli-triggered drug delivery systems which utilize CO_2_, bicarbonate or pH changes as signaling molecules. This can improve the efficiency of these antidote based delivery systems (Satav et al., 2010).

### Blood substitutes

The current blood substitutes have inadequate CO_2_ removal rate, which leads to coma or death. CAs can be utilized along with catalase (CAT) and superoxide dismutase (SOD into the PolySFHb substitute (PolySFHb-SOD-CAT-CA) in order to overcome this limitation (Bian et al., 2011).

## Conclusions and future perspectives

CAs are a class of enzymes which are essential for the survival of living beings. This enzyme not only helps in our metabolic activities but also aids in the protection of Mother Nature. Due to anthropogenic activities, the nature is getting destroyed day by day. Global warming is having its toll on the climate and weather. Carbonic anhydrase can aid in tackling the future catastrophes due to global warming. Unfortunately the CCS and biomineralization techniques are either in the lab stage or pilot plant stage. Researchers are constantly attempting to address a few of the key issues related to this technology, thus, it has become quite difficult to generate a public consensus on CCS technology. The technology needs to be ameliorated. We are also not able to bring down the carbon emissions as fossil fuels are (also will be in near future) the main energy source which drives today's world. Although much is known about CAs, the current metadata reveals the presence of many CA genes in the extreme realms of earth which are waiting to be discovered. These are mostly from unculturable microbes. Bioinformatics tools have been useful in identifying different CA encoding genes. Once identified, they can be easily cloned and expressed in different microbial hosts for studying their novel properties and utility in CO_2_ biomineralization. Currently the unexplored data is so vast that we may discover a few novel classes of CAs in addition to the known six classes in future. CAs from polyextremophilic microbial sources have already been tried for CCS related strategies. Enzyme immobilization techniques permit the repeated use of the enzyme and designing continuously operating reactors. We need to improve the CA immobilization technology so that it becomes cost effective and readily accepted by people. Further research efforts are called for developing highly efficient and robust CAs, efficient immobilization techniques and designing continuously operating reactors for cost effective biomimetic carbon sequestration.

## Author contributions

All authors listed have made a substantial, direct and intellectual contribution to the work, and approved it for publication.

### Conflict of interest statement

The authors declare that the research was conducted in the absence of any commercial or financial relationships that could be construed as a potential conflict of interest. The reviewer SD and handling Editor declared their shared affiliation, and the handling Editor states that the process nevertheless met the standards of a fair and objective review.

## Footnotes

^1^Emission database for global atmospheric research EDGAR, European Commission. Available online at: http://edgar.jrc.ec.europa.eu/ (Accessed February 27, 2017).
